# Is the Consumer Ready for Innovative Fruit Wines? Perception and Acceptability of Young Consumers

**DOI:** 10.3390/foods10071545

**Published:** 2021-07-04

**Authors:** Valentina Maria Merlino, Daniela Fracassetti, Alessandra Di Canito, Simona Pizzi, Danielle Borra, Nicole Roberta Giuggioli, Ileana Vigentini

**Affiliations:** 1 Department of Agricultural, Forest and Food Sciences (DISAFA), University of Turin, 10095 Turin, Italy; danielle.borra@unito.it (D.B.); nicole.giuggioli@unito.it (N.R.G.); 2 Department of Food, Environmental and Nutritional Sciences (DeFENS), University of Milan, 20133 Milan, Italy; daniela.fracassetti@unimi.it (D.F.); alessandra.dicanito@unimi.it (A.D.C.); simonaapizzi@gmail.com (S.P.); ileana.vigentini@unimi.it (I.V.)

**Keywords:** consumer expectation, fruit wine, new product acceptability, new entries, neophiles, Z generation, Millennials

## Abstract

The consumption of traditional wine has decreased in Europe during the last fifteen years. In parallel, new wine alternatives obtained by blending wines and fruit juices or by flavoring wines with artificial or natural flavors have appeared on the market. Recently, an innovative fruit wine obtained by co-fermentation of grape must and kiwi juice has been proposed and its potential of attraction for consumers should be exploited. To assess the potential consumer acceptability and expectations towards this new product, an online choice experiment has been conducted involving a consumer group of young adults (18–35 years old; *n* = 373). After the data collection, participants were divided into two groups according to whether they had already tasted a fruit wine (neophiles) or had never tasted it (new entries). For each group, the individual’s responses (on wine consumption habits, expectations and willingness to consume and pay a fruit wine) were analyzed through Principal Component Analysis. Different consumption styles and expectation patterns were defined in the two groups. However, in general, neophiles showed consumption patterns based on the evaluation of fruit quality, sales format, alcoholic content and the presence or not of bubbles, not giving importance to the brand. In contrast, new entries’ responses identified consumption patterns driven by the willingness to pay for a new product, the product value for money and packaging features. Differences between the two groups in expectations about the product sensory characteristics also emerged. These findings should contribute to this area of study by integrating environmental, economic and social dimensions and addressing food innovation and sustainability in the fruit and wine chains.

## 1. Introduction

Sustainable development refers to a “development that meets the needs of the present without compromising the ability of future generations to encounter their own needs”; it faces economic, social and political activities with potential impacts on the environment [1]. In this context, agriculture plays a central role since a large amount of resources and inputs are used to produce food. These factors can significantly affect the environment and the food safety of the products themselves with consequences that can be harmful to the planet (i.e., accumulation of pesticides, soil erosion, gas emissions). Thus, new approaches and solutions are needed to address worsening of these problems [2].

### 1.1. Food Sustainability and Food Waste

The concept of food sustainability is strongly linked to that of food waste. Waste concerns all the phases of the agri-food system (field production, transformation, storage, distribution and final consumption) with methods and for specific causes that are different for each phase. A form of waste is represented by the surpluses, i.e., all those products that, while meeting the quality standards of food safety, are not purchased and/or used in the transformation processes for different causes [3]. About 5.6 million tons of food surplus is produced per year in Italy, corresponding to about one-sixth of what is consumed. This means that the food is produced, transformed, and distributed but, for several reasons, it is not sold or consumed [4]. Regarding fruits and vegetables, the Food and Agriculture Organization (FAO) of the UN has estimated that 21.6% are lost worldwide [5]. Recently, the FAO has launched the UN’s International Year of Fruits and Vegetables 2021 with an appeal to improve healthy and sustainable food production through innovation and technology and to reduce food loss and waste [6]. In Italy, about 3% of the agricultural products are not harvested (10% is fresh fruits), causing great damage in environmental and economic terms [7]. Indeed, when the supply is greater than the demand, farmers prefer to leave still edible products in the field, because it is more cost convenient. This situation can increase the risk of plant diseases, determining the increase in chemical inputs consequently and, again, costs and impacts on the environment.

### 1.2. The Fruit Production Surplus (Food Waste) and the Wine Industry

The surplus of fruit production represents a production waste. In Italy, fruits with a high probability to generate waste include, among others, kiwi fruits and grapes. Regarding kiwis, Italy is the second highest producer in EU and the third in the world, with a market of 435,000 t per year, which generates a commercial surplus of EUR 343 million [8,9]. Regarding grapes, Italy is the second highest producing country in the world; Italy and other EU member states account for about two-thirds of global production [10]. Of the estimated 182.7 million hectoliters of produced grape must in the EU-28 in 2018/2019, nearly 97% was used for wine production [11]. However, while the global grape and wine production increased in 2018, the wine consumption was stable [12]. Moreover, the wine industry underwent a loss of about EUR 5.4 bn in the first quarter of 2020 due to the unsold products as a result of the COVID-19 pandemic [13,14]. Thus, if grape production itself does not generate a fruit surplus, the wine sector often finds itself managing relevant quantities of unsold wine which becomes production waste. This situation does not guarantee a fair remuneration and creates/reinforces an imbalance between the phases of the supply chain, reducing the market power more and more on the demand side. Moreover, in terms of food sustainability, the wine chain follows the traditional linear extract-produce-use-dump material and energy flow model [15]. This one-way economy is unsustainable and useful practices must be implemented to create a system for valorization of surplus [7,16]. To this aim, the introduction of innovative and sustainable strategies, which find new uses for fruit and have the highest potential to limit food loss and wastes, could represent a strategic tool for the creation of additional value in the supply chain. The wine world is going through a rapid transformation linked to changes in consumer preferences, consumption habits, climate, new regulations, and the reduction in available economic resources. In addition, the differentiation of production can represent a marketing tool for producers to achieve new consumer segments and markets [17,18]. People are more and more attracted by innovative low-alcohol beverages, aromatically pleasant and naturally enriched in bioactive compounds. According to these standards, new alternatives of wines have appeared on the market [19,20,21]. Those beverages are obtained by blending wines and fruit juices or flavoring wines with artificial or natural aromas and have medium alcoholic contents (from 8 to 10.5% (v/v)). Several fruits other than grape have been used throughout history to produce alcoholic beverages. Over time, the consumption of these products by local communities has given rise to recipes for homemade fruit wines that have been handed down through the generations [22]. Recently, an innovative fruit wine has been proposed obtained by co-fermenting grape must and kiwi juice [23], whose potential attractiveness to consumers should be exploited. The introduction of these products in the market could therefore be highly dependent on consumers’ attitudes to enjoy trying new beverages or foods; this last psychological condition is called neophilia being the opposite of neophobia or adventurousness [24].

### 1.3. Research Aims

Considering young consumers as a target more attracted and willing to taste new products [18,25,26], this research was based on a survey about young people’s expectations (Millennials and Z generation) and the evolution driving the acceptability and appreciation of fruit wines. For this purpose, a choice experiment was conducted by involving a sample of young consumers divided in neophiles, who had already tried a fruit wine, and in new entries, who have never tasted fruit wine. For each group, a Principal Components Analysis (PCA) was performed in order to determine the effect of expectations, perceptions and willingness to pay on different established consumption orientation patterns. These research findings will contribute to understanding the potential impact on the market of fruit wine integrating environmental, economic and social dimensions and addressing mutually food innovation and sustainability in both fruit and wine chains.

## 2. Materials and Methods

**Figure 1:**
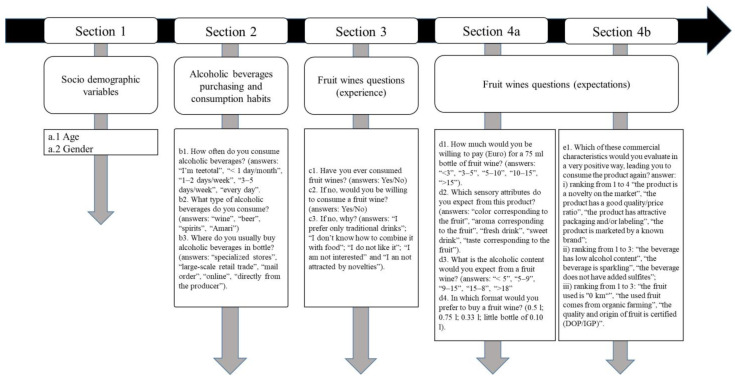
Questionnaire theoretical framework. The arrows indicate the logical path the respondents had to follow during the questionnaire filling process.

The data were collected through an online survey created with Google Forms (Google Inc., Mountain View, 94043, CA, USA) and disseminated by e-mail and social networks in 2020. The survey was conducted in accordance with the ethical standards set out in the Declaration of Helsinki. Participation of all respondents was voluntary and informed consent was provided by all respondents. Completion of the questionnaires was not rewarded and only 10% of all questionnaires sent were not completed. The questionnaire was structured by different sections, as described in Figure 1.

The developed questions submitted in the surveys were open ended (a1, a2), closed-ended (c1, c2), and double check-all-that-apply (CATA) questions (Section 2, question c3 of Section 3, Section 4a), the latter of which used to investigate on individual’s alcoholic beverages consumption and purchasing habits [27,28]. Questions of Section 4b were structured in order to understand which characteristics of fruit wine could be the most important ones with the objective of building up a product prototype in line with individual’s expectations. For this purpose, three questions were proposed and, for each of them, the respondents had to rank the features they would like in the product according to their preferences. The surveys’ answers were processed to define the expectation and the perception of fruit wine from two individual groups: the first one defined by neophiles who declared (question c1, Figure 1) they have already tasted a fruit wine and the second formed by new entries who had never tasted this type of product. The differences in consumer’s expectations about fruit wines in terms of sensory features between the two groups were tested using the Chi-square test [28]. The principal component analysis (PCA) was performed using SPSS 27.0 software (SPSS Inc., Chicago, IL, USA) package for Windows, selecting the answers to Sections 4a and 4b of the two groups separately with the aim to define different consumption orientation based on consumer experience, expectation and perception towards a new product. In fact, the PCA, or proper orthogonal decomposition, is a data simplification technique used in multivariate statistics to reduce the greater or lesser number of variables describing a data set to a smaller number of latent variables, limiting the loss of information as much as possible. In our research, the factor extraction was based on Varimax rotation. An initial correlation matrix was used for this study considering the minimum correlation value (loadings) higher than 0.3. Sample adequacy was analyzed using the Kaiser-Meyer-Olkin (KMO) index, and Bartlett’s sphericity test was performed to test the hypothesis that the correlation matrix corresponded with the identity matrix.

## 3. Results and Discussion

**Figure 2:**
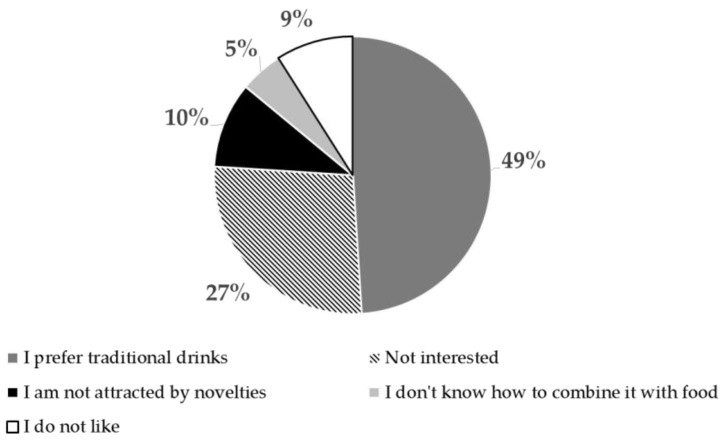
Consumers’ motivations for not consuming fruit wines (*n* = 119).

In the present study, the interviewees were 118 neophiles, 253 new entries and only 2 persons declared they were teetotal (who stated that they choose or are characterized by abstinence from alcohol). Neophiles include respondents that have already tried fruit wines as they answered “yes” to the c1 question (Figure 1), whereas the new entries group has not experienced the product yet. The first group was balanced in terms of gender, while the second accounted for a major number of male (66%) with respect to the female (44%). Both groups were equally represented by individuals from the Z generation (about 60%) and Millennials (about 40%). Concerning the whole sample, 68% of individuals stated they would be willing to consume a fruit wine in the future, whereas 32% (*n* = 119) indicated they would not like to try this new product. Considering the latter consumers, the percentages of responses obtained regarding the reasons why they do not intend to approach this new product (question c3 in Figure 1) are shown in Figure 2. As reported in this graph, neophiles (9%) also answered. On the contrary, the principal motivation was the individual preferences towards traditional drinks (e.g., wine, beer and spirits), followed by the absence of interest and attractiveness towards product innovation.

Significant differences emerged when the consumption habits of alcoholic beverages between the two considered groups were compared, especially in the case of spirit and beer consumption. In particular, the new entries category was more oriented towards spirit consumption, while the neophiles cluster constituted regular beer consumers (Table 1).

In terms of consumption frequency of alcoholic beverages, the neophiles and new entries statistically differed considering the most frequent consumption level (every day), highlighting a higher propensity of consumption in the new entries (Table 2).

Finally, the two groups were also different regarding the purchase place of alcoholic beverages; the new entries showed the strongest preferences for the large-scale retails, while the neophiles were more oriented towards the producers and the specialized shops (Table 3).

### Consumers Expectations and Perception: Neophiles vs. New Entries

The expectations about the sensory characteristics of fruit wine changed within the two groups (Table 4). In particular, if the neophiles did not expect a scent, color and taste corresponding to the original fruit, as well as a fresh drink, these latter features were awaited by the new entries. Indeed, neophiles have familiarity with this kind of beverage and the expectancy effects generated by the fruit wine consumption could have built up in their minds to remind them of previous experience(s) not only linked to extrinsic properties but also to intrinsic cues of the product [29]. In some fruit wines, a disconfirmation between the expected taste experience and the actual one could appear; this could depend on the type of fermented fruit or the applied fermentative and technological strategies which introduce variability in the aroma profiles of wines making the original scent of fruit poorly represented. The maturity of fruit also affects the overall perception of fruity note as fruitiness can be more evident when slightly overripe fruit is fermented. On the contrary, if the fruit is collected earlier, other notes are dominant as can occur in pear wines produced with unripe fruit where a typical apple taste could be dominant [30]. The wine produced from kiwis, for example, is described as of unusual composition and Riesling Sylvan character [31]. Differences can be found for both volatile and non-volatile components in mango wine based on the sugar content of juice used for the production of wine [32]. The harvest of fruits represents an important aspect affecting the composition of the grape itself [33]. Not only this, but this effect of expectation taste disparity can take place in wines produced with neutral grapes (e.g., Chardonnay), where tropical and citrus notes can appear depending on the technology applied (e.g., addition of Gewürztraminer grape marc) [34]. On the contrary, individuals classified as new entries expect a product that is more sensory related to the derived fruit. This result agrees with the literature, confirming that the typical characteristics of the fruit (in our case the scent, freshness and taste of kiwi) are expected in the derived wine [35,36,37]. In general, different research studies have emerged on how the consumer familiarity with fruit can influence expectations regarding the sensory characteristics of the resulting drink or product. For example, knowledge of the color of the fruit, which is then reflected in the color of the drink, can determine the degree of perception of certain sensory characteristics (bitterness, aroma, sweetness, etc.) of the resulting drink [35]. In the same reference, Gous et al. (2019) demonstrated the aroma of grapefruit-like model beverage could influence the perception of basic tastes and vice versa [35,36]. The authors found that specifically for the new entries, their expectations did not coincide with the real characteristics of this beverage. This remarkable result could create a risk condition of real acceptance of the drink not conforming to the expressed expectations. However, both the two consumers groups were in accordance with the expected sweetness of the fruit wine [35,37]. This latter aspect could be an advantage in terms of the marketability potential of wines produced from fruit; the carbohydrates contained in fruit represent a source of energy and are often associated with sweetness, an intrinsic characteristic with a powerful hedonic appeal [38].

Among the neophile consumers, four main components were distinguished, defined by the declared expectations on the new product (Table 5). PCA explains 63% variance among the four components. The first one (21.9% of the total explained variance) is defined by the expectation to find the new product in a big format (positive correlation) and to all the variables linked to the intrinsic product characteristics that could define fruit wine on the market. A negative correlation emerges with novelty and the known brand, since these individuals have already tested the product, but a positive one with the quality/price ratio. This component defines a consumption model expectation towards a not sparkling product, for which is not important the alcoholic degree, with the absence of sulfites, derived from quality fruit, not organic, but certified [39]. The second component (15.8% of the explained variance) is different from the first one because the importance of relying on a known brand for a new product is demonstrated, distinguished by the new label and a good quality/price ratio. In this case, this consumption pattern is based on the acceptability of the new product, if guaranteed by a known brand [40,41]. Thus, this component profiles an attitude based on the brand loyalty value [42,43]. Additionally, in this case emerged as important the certified quality of the fruit used for the beverage production. The third component (13.9% of the explained variance) is defined by the expectations of the product (positive correlation) relative to the format and alcoholic content. Moreover, it defines a model of choice with regard to the product focused on the external aspect (label) and on certain chemical and sensory attributes, such as the low alcoholic content and the production of a still drink, as this component does not emerge as important with regard to fruit quality. The last component (11.4%) is again unrelated by the importance of the brand knowledge, while a high alcoholic content is expected, which defines a sparkling product. In general, this first analysis based on the preferences of neophiles revealed attitudinal profiles based mainly on sensory expectations and the product on the market, but not on the willingness to pay for a new product.

In the case of new entries, four components of product expatiation were defined (Table 6). The first component (17.8% of the total explained variance) defines an expectation model about fruit wine above all to the type of product on the market (reassuring because of a well-known brand, with attractive packaging and good value for money). The other variables are also significant with positive loadings that lead back to a proactive profile that recognizes, in this product, from certified quality fruits, the importance of the alcoholic content, the presence of bubbles and no sulfites added. The particular importance of alcoholic content is in line with the frequency of alcoholic beverage consumption of these individuals. In general, this orientation pattern highlights few expectations of the product other than those deriving from their knowledge of the fruit and would only try the new product if it was linked to a known brand. Therefore, it was found that unfamiliarity with the new product determines an acceptability based only on features that induce confidence (such as the brand name) [40,41] or desirable features in relation to familiarity with the raw material [44]. Instead, the second component (15.0% of the explained variance) is defined by a greater propensity to spend money for this new product, with attention on the product format and alcoholic content. Therefore, the consumer intention about the new product is demonstrated for this component by the declared willingness to pay (WTP), as well as expectations about the format. Indeed, as shown in Grasso and Asioli (2020), the study of WTP for new products, as well as extrinsic characteristics, reflects consumer acceptability and helps industries to suggest a market price in line with consumer needs [45]. The brand is not synonymous with quality and safety for these individuals that aim, instead, for attractive packaging and a good value for money. They focus their attention on the extrinsic quality attributes of products, probably because they are still unaware of the product’s intrinsic characteristics [46]. In fact, they do not resemble the intrinsic characteristics of the product, but believe in the correlation between the quality of the fruit and the final product. This result confirms how consumer acceptance is driven by risk perception and by the perception of the potential benefits [47]. In addition, it has been proved that consumer knowledge and experience of a product influence product quality evaluation and choice [48]. The assessment of those characteristics, which are directly manageable and accessible to consumers (in this case the product extrinsic characteristics), is related to an individual’s unfamiliarity with a new product [49]. It has also been shown how the quality of the raw material and the safety of the production process affect the assessment of the quality of the final product [50]. The third component (13.9% of the explained variance) is defined above all by the characteristics of the product and the certified quality of the fruits. Finally, the latter component (11.3% of the explained variance) is positively correlated to the willingness to pay, even if the image of the product is not so attractive. The major characteristics considered were a product obtained from organic farming (probably safer) with a low alcoholic content. Given that high neophobia is correlated with low or no WTP [51], it would be important for a proportion of new entries to differentiate the product communication program by focusing on aspects that emerged as significantly positive in defining their choice orientations.

## 4. Conclusions

The survey carried out on the consumer perception of a novel fruit wine demonstrated that the marketplace could be attracted to and potentially ready for this innovation. The sustainability aspects that distinguish this product along with the social impact of a low alcoholic drink, however, obtained from a grape must may represent a further economic fly wheel in the alcoholic beverage market. The differences in consumer perception, considering individual previous experience, could represent a marketing tool to consider in the product communication strategies. Our findings will help the beverage industry to make a proper communication plan related to the drink characteristics in order to reach and satisfy the expectations of consumers. In particular, this last statement takes on greater importance for the case of new entries who, in this research, expressed sensory expectations for the new product that could not change in several fruit wines. In this case, therefore, this result makes it possible to adequately highlight the descriptors affecting the acceptability of the new entries. A capillary communication of product characteristics, as well as its impact of society and the environment, can be a successful strategy for its introduction to the market. This communication plan could also attract the interest of the neophiles, highlighting the strengths of fruit wine. However, we recognize some limits of the research related to the sample composition and size. This limit may be overcome by extending the research in other Italian regions and areas to compare different consumer profiles, especially considering more age groups of individuals. Nevertheless, from our results, the commercialization of fruit wine could have a promising future and they could represent an effective strategy to limit food losses and wastes, improving the sustainability of fruit and wine chains.

## Figures and Tables

**Table 1:** Consumption habits of alcoholic beverages for new entries (*n* = 253) and neophiles (*n* = 118).

Product	New Entry	Neophiles	Chi-Square	*p*-Value
Spirits	19%	15%	21.398	***
Wine	42%	41%	2.465	0.119
Amari	1%	0%	1.549	0.214
Beer	38%	44%	11.49	**

The *p*-value refers to the statistical significance level: *** *p* < 0.001, ** *p* < 0.01.

**Table 2:** Consumption frequency of alcoholic beverage for new entries (*n* = 253) and neophiles (*n* = 118).

Frequency	New Entry	Neophiles	Chi-Square	*p*-Value
<1 day/month	15.10%	16.00%	4.338	0.508
1–2 days/week	39.10%	39.50%	4.658	0.702
3–5 days/week	30.40%	35.30%	1.203	0.506
every day	15.40%	9.20%	4.345	**

The *p*-value refers to the statistical significance level: ** *p* < 0.01.

**Table 3:** Purchase place of alcoholic beverage for new entries (*n* = 253) and neophiles (*n* = 118).

Place of Purchase	New Entry	Neophiles	Chi-Square	*p*-Value
Large-scale retail trade	45.50%	30.00%	3.650	*
Mail order	3.60%	1.40%	3.642	*
Online	2.00%	4.00%	0.449	0.503
Directly from the producer	25.10%	33.90%	0.101	*
Specialized stores	23.80%	30.60%	0.985	0.997

The *p*-value refers to the statistical significance level: * *p* < 0.05.

**Table 4:** Expectations of new entries (*n* = 253) and neophiles (*n* = 118) in terms of sensory characteristics of fruit wine.

Which Sensory Attributes Do You Expect from This Product?	Answer	New Entries	Neophiles	Chi-Square	*p*-Value
Color corresponding to the fruit	Yes	12.6	22.2	5.625	*
	No	87.4	77.8		
Scent corresponding to the fruit	Yes	57.1	26.8	4.472	*
	No	42.9	54.7		
Sweet drink	Yes	15.4	17.9	0.398	0.528
	No	84.6	82.1		
Fresh drink	Yes	40.9	28.2	5.582	**
	No	59.1	71.8		
Taste corresponding to the fruit	Yes	56.3	39.3	9.245	**
	No	43.7	60.7		

*p*-value significant level: * *p* < 0.05, ** *p* < 0.01.

**Table 5:** Fruit wine expectations and perception profiles by neophiles.

Variables	Neophiles (*n* = 118)					
	Mean	SD ^1^	Components			
			1	2	3	4
Product expectations						
Willingness to pay (for a 750 mL bottled fruit wine)? (0–4)	2.01	0.663				
In which format would you prefer to buy a fruit wine? (0–3)	2.66	0.579	0.496		0.579	
What is the alcohol content would you expect from a fruit wine?	1.72	0.842			0.559	0.520
Which of these characteristics would you rate extremely positively leading you to still consume the product?						
Characteristics of the product on the market						
The product is a novelty on the market (1–4)	2.72	1.218	−0.451	−0.578		
The product has a good quality/price ratio (1–4)	2.47	1.205	0.701			
The product has attractive packaging and/or labeling (1–4)	2.66	0.740		0.432	0.488	
The product is marketed by a known brand (1–4)	2.14	1.174	−0.330	0.519		−0.490
Intrinsic characteristics of the product						
The beverage has low alcoholic content (1–3)	1.84	0.767	−0.534		0.378	−0.538
The beverage is sparkling (1–3)	2.14	0.795	−0.392		−0.538	0.552
The beverage does not have added sulfites (1–3)	2.01	0.872	0.827			
Characteristics of fruits						
The used fruit is “0 km” (1–3)	2.03	0.848		−0.828		
The used fruit comes from organic farming (1–3)	2.08	0.726	−0.458	0.471		0.347
The quality and origin of fruit is certified (PDO/IGP) (1–3)	1.89	0.873	0.534	0.413	−0.395	

^1^ Standard Deviation.

**Table 6:** Fruit wine expectations and perception profiles by new entries.

Variables	New Entries (*n* = 253)					
	Mean	SD ^1^	Components			
			1	2	3	4
Product expectations						
Willingness to pay (fora 750 mL bottled fruit wine)? (1–3)	1.60	0.804		0.319		0.382
In which format would you prefer to buy a fruit wine?	2.28	0.882		0.469	0.465	
What is the alcohol content you would expect from a fruit wine?	1.31	0.935		0.476		
Which of these characteristics would you rate extremely positively leading you to still consume the product?						
Characteristics of the product on the market						
The product is a novelty on the market (1–4)	2.53	1.137	−0.397	−0.548		
The product has a good quality/price ratio (1–4)	2.22	1.131	−0.476	0.544		
The product has attractive packaging and/or labeling (1–4)	2.56	0.980	0.451	0.534		−0.381
The product is marketed by a known brand (1–4)	2.69	1.172	0.371	−0.440		
Intrinsic characteristics of the product						
The beverage has low alcoholic content (1–3)	1.90	0.730	0.620		0.350	0.247
The beverage is sparkling (1–3)	2.11	0.767	0.449		−0.479	−0.566
The beverage does not have sulfites added (1–3)	1.98	0.931	−0.856			0.273
Characteristics of fruit						
Fruit used is “0 km” (1–3)	2.02	0.838		0.454	−0.689	
The fruit from which it is produced comes from organic farming (1–3)	2.00	0.744	0.438			0.656
The fruit for production is certified (DOP/IGP) (1–3)	1.98	0.869		−0.343	0.710	−0.546

^1^ Standard Deviation.
